# Hallucinations: Or the Rational History of Apparitions, Visions, Dreams, Ecstacy, Magnetism, and Somnambulism

**Published:** 1853-12

**Authors:** 


The works noticed below came too late for notice in our department of Reviews. Several others will receive early attention:

*Hallucinations: or the Rational History of Apparitions, Visions, Dreams, Ecstacy, Magnetism, and Somnambulism.* By A. BRIERRE DE BOISMONT, Docteur en Medecine de la Faculté de Paris, etc., etc., etc. Philadelphia: Lindsay & Blakiston.

Here is an octavo devoted to the consideration of those minor forms of insanity enumerated in the title. We say minor forms of insanity, but the leading idea of the book is to prove that certain hallucinations are consistent with a sound mind.

Our readers are not to suppose that this volume is one of the new-light productions of the day. It is strictly a medical work, and one evincing great learning and research. It is to be classed with the works of Abercrombie, Walter Scott, and Brewster, as a treatise on mental philosophy; while to the physiologist and physician it has the higher merit of being founded on correct scientific knowledge, and devoted to the treatment and cure of these maladies.

The work is divided into twenty chapters, of which twelve are devoted to the history of the different forms of hallucination; and the remainder to their pathology, treatment, and their religious and legal bearings.

It is in the second chapter that the idea which has encountered most opposition is enunciated. The author here argues the *coexistence of certain hallucinations with unimpaired reasoning powers.* These illusions may be either recognized by the patient, and corrected by his reason, or they may be unrecognized as errors, and uncontradicted by the reason. Instances of the former are very common. They are generally errors of perception of some one of the special senses; more frequently that of sight. All imaginative persons have in some measure the power of reproducing the images of persons and things previously seen; and in the condition called reverie, which is most favorable to their production, it is very easy to people the darkness of a room with forms and faces such as no mortal eye ever saw, and no artist could paint. And this is really an error of the sense of sight: the objects are actually beheld, the impression on the retina being made from within outward, and not as usual by rays of light reflected from an object.

The very common habit of gazing into a bed of living coals, and building up among them palaces and cities of the most gorgeous character, is perhaps the earliest approach to the condition of hallucination. Without stopping to trace the intermediate grades of this first stage, we will instance the case of the judicial functionary mentioned by Sir Walter Scott. This man, distinguished for mental acuteness, and occupying a high office which he filled with great ability, was followed by a spectre, first in the form of a cat, next as a gentleman usher, ridiculously dressed, and everywhere preceding and announcing him; and finally, as a skeleton, ever walking by his side. The patient was conscious that these appearances were unreal, and so careful was he of his reputation as a jurist, that he never revealed his affliction to any one save his physician. Still with this consciousness of error of perception strong upon him — with the knowledge and belief that his senses were deceived, and with his reasoning powers unimpaired, this strong minded man was worried to his grave by these unwelcome companions.

Was this in any degree insanity? M. De Boismont says not; and we are inclined to share in his belief. The distinction, (useful in argument, if not strictly true in point of fact,) should here be made between reason and perception. Perception furnishes us our facts, and from them our reason draws its conclusions. In the case of the judge, he was so entirely conscious of an error of perception, that his reason took no cognizance of the false appearances presented. Yet had he allowed others to know his condition, it is very probable that his reputation as a jurist, and his interests as a man, would have suffered from unjust suspicions of his sanity.

But there is still another class of cases where the error of perception is not corrected by the reason. M. De Boismont gives many such cases, when the hallucination was considered by the patient as something supernatural, and to be thoroughly believed. Such hallucinations are common in great men, men filled with some great idea: as the conduct of a revolution, social, political, or religious. Luther saw the devil; Constantine, the cross; Bonaparte, his guiding star; Joan of Arc, her angel visitants; and even the demagogues of the reign of terror were subject to such deceptions.

This class of cases differs from the preceding, in the fact that the illusion is unrecognized, and to its existence we must attribute the expression of the poet:

"Great wits to madness nearly are allied."

But why, asks our author, should the reputation of these great men — many of them eminently good men — be stained with a reputation of insanity, when their actions are all marked by a degree of foresight, and power of reason and combination, which is the highest evidence of sanity? Here is merely an error of perception. The reason believes it, but does not act imprudently upon it.

One great error in the consideration of insanity, lies in the non-recognition of the fact that the mind is a unit. The doctrines of the phrenologist are subversive of all moral restraint or responsibility. A multitude of different faculties or organs, packed in the cranium like so many eggs in a basket—one of which may be addled without impairing the integrity of the others—relieves the possessor of them of all accountability. The distinction is lost between the subject and the object—between the mind which governs, and the brain which is the instrument. In contradiction to this idea, M. De Boismont asserts the perpetual integrity of the mind. Like the king in the game of chess, it is inviolable. The brain itself may be disordered; the workings of fever or delirium, the action of opium, or the destructive processes of organic disease, may impair the tone, or destroy the integrity of the instrument; but the mind still lives unimpaired, as in a case which we have known, where one who for many years had been a maniac, recovered her reason while dying from phthisis pulmonalis, and for the fortnight preceding her death, was, in every sense of the word, sane.

We find that our interest in this subject has betrayed us into a longer sketch than we had intended. We assure our readers that this book will repay their perusal of it.

For sale by Derby, Orton & Mulligan. S. B. H.

